# Parkinson's Disease as Illness Anxiety Disorder: A Rare Case Report

**DOI:** 10.1002/ccr3.70973

**Published:** 2025-09-26

**Authors:** Ronak Fatahi, Mohammad Sadegh Khooshab, Seyede Fatemeh Sadatakhavi, Amin Ziyaei, Reza Bidaki

**Affiliations:** ^1^ School of Medicine Kermanshah University of Medical Sciences Kermanshah Iran; ^2^ Student Research Committee Shahid Sadoughi University of Medical Sciences Yazd Iran; ^3^ Department of Psychology Yazd Branch, Islamic Azad University Yazd Iran; ^4^ Department of Psychiatry, Research Center of Addiction and Behavioral Sciences Non‐Communicable Diseases Research Institute, Shahid Sadoughi University of Medical Sciences Yazd Iran

**Keywords:** anxiety, hypochondriasis, illness behavior, Parkinson's disease

## Abstract

Illness anxiety disorder (IAD) is a psychiatric condition characterized by excessive fear and anxiety about developing a serious illness. Individuals with IAD constantly search for signs of serious illness, despite negative test results. This case report presents a patient with IAD who was concerned about developing Parkinson's disease. A 37‐year‐old married woman was worried about developing Parkinson's disease. Same as her mother, she had an anxious personality, albeit there was no history related to Parkinson's disease in her family, and all physical examinations were normal, but she exhibited a hand tremor. After the second pregnancy, she experienced depression, and after her father's death, the situation got worse. There are rare cases of illness anxiety disorder (IAD) related to other neurodegenerative disorders like multiple sclerosis in the literature review, but we believe this is one of the first and very rare reports regarding a patient with illness anxiety disorder who believes in involving Parkinson's disease, and it progresses. We are of the opinion that treatment for this disorder can be more useful to eliminate overvalued ideation and treat the patient.

## Graphical abstract

Cognitive‐behavioral model of Illness Anxiety Disorder highlighting symptom misinterpretation and behavioral consequences.

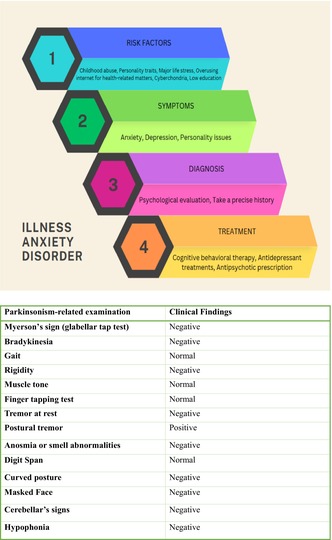

**Abbreviations**
CBT: cognitive behavioral therapy
CTA: computed tomography angiography
DDX: differential diagnosis
FND: functional neurological disorder
IAD: illness anxiety disorder
MDD: major depressive disorder
SSRIs: selective serotonin reuptake inhibitors
TCAs: tricyclic antidepressants

**Summary**

- Although Parkinson's disease has hard treatment, in some cases it can be managed.
- In this rare report, a patient with an illness anxiety disorder may exhibit symptoms of Parkinson's disease, including fear of immobility, extreme tremors, and disability.
- Physicians can assist patients in preventing unnecessary tests and investigations by assuring them that their symptoms do not correspond to Parkinson's disease.

## 1 Introduction

**FIGURE 1:**
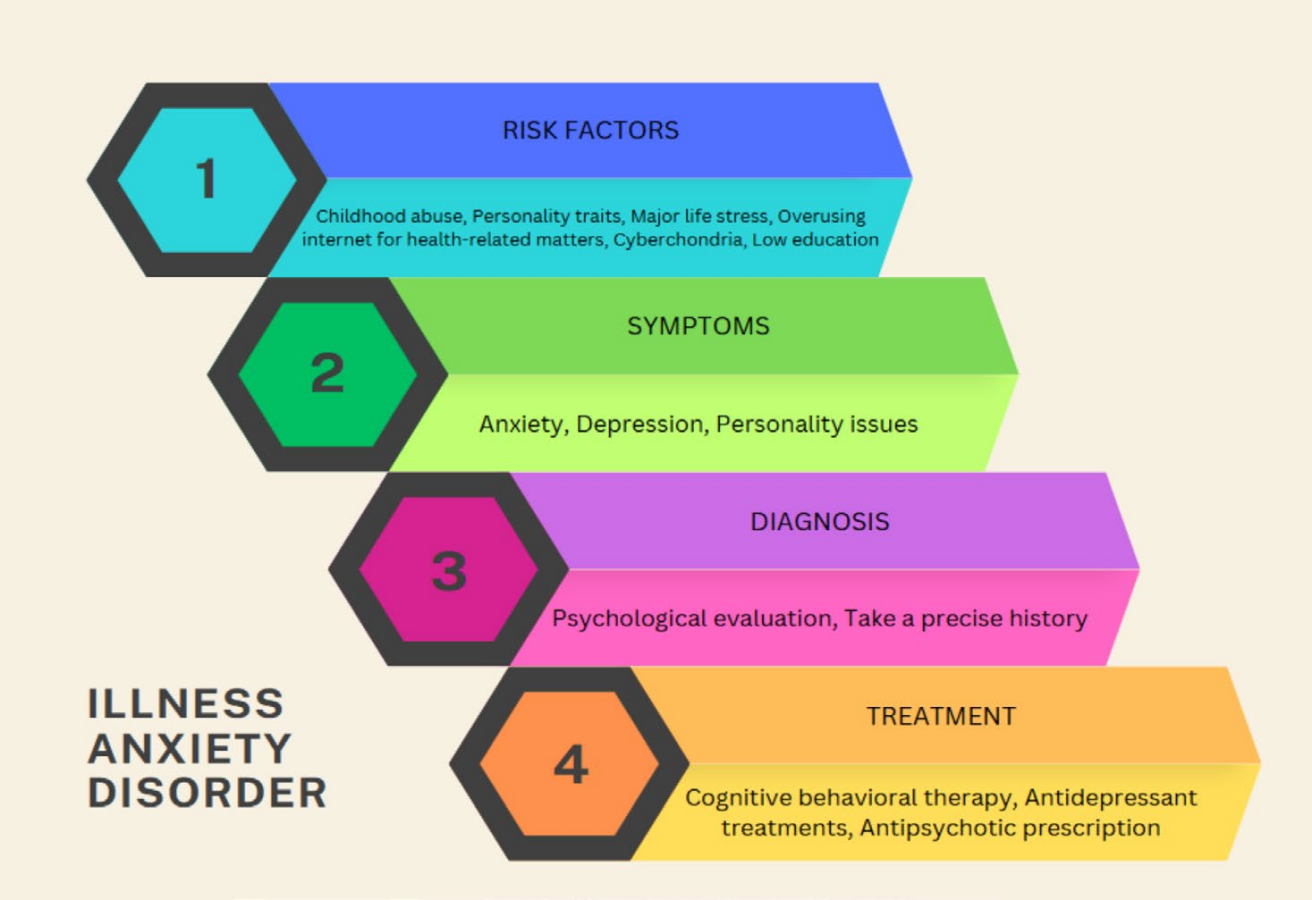
Overview of illness anxiety disorder.

Illness anxiety disorder (IAD) (previously known as hypochondriasis) is a mental disorder that is defined by a high level of fear and anxiety about having or developing a serious illness [1]. Individuals with IAD constantly search for signs of serious illness, even when medical evaluation and examination are normal [2]. This disorder can significantly impact an individual's quality of life [3]. Even though depression and anxiety disorders tend to be more common in females, IAD impacts men and women equally [4, 5]. This disorder has two types: the “care‐seeking type” of this disorder is characterized by frequent medical care and diagnostic procedures, while the “care‐avoidant type” is characterized by avoidance of medical care [3]. The diagnosis of IAD needs 6 months of extensive care‐seeking behaviors, no evidence of physical signs and symptoms, and a concern about the medical condition [6]. Typically, this condition begins in early or middle adulthood and worsens with age [3], some evidence suggests that it may begin in early childhood [7]. In some rare cases, this disorder may be demonstrated by anxiety from developing appendicitis in a patient who had an overvalued ideation about having appendicitis, but her examinations by surgeons and gastroenterologists did not reveal anything [8], getting covid‐19 especially after the covid‐19 pandemic [9] or in the context of pulmonary thromboembolism and rabies [10, 11]. Physicians should form a therapeutic relationship with their patients to assist them in dealing with their fears, which is the basis of treatment for individuals [1]. Physicians need to provide reassurance to these patients in order to treat them effectively [12]. Other treatments include cognitive behavioral therapy, attention training, and commitment therapy [13]. Studies support that fluoxetine is safer and more effective for these patients [13]. This disorder's characteristics are summarized (Figure 1). In this study, we report a case of illness anxiety disorder in a patient who was concerned about developing Parkinson's disease.

## 2 Case History/Examination

A 37‐year‐old married woman, who was right‐handed, unemployed, and with low socioeconomic status, had a math diploma and was worried about getting Parkinson's disease. The symptoms got started 8 months before the presentation, and she described how her thumbs were shaking while using her cell phone. She sought Google because she deemed there was a meaningful connection between such symptoms and Parkinsonism. The woman got married and had 2 boys. Her marriage dates back about 10 years, and during the 6 years they were childless, this led her to become more anxious. She experienced depression after the first childbirth, clarifying that she felt like it was the end of the world during these years. Before the second pregnancy, not only did she have an obsession, but she also felt guilt. Moreover, in the 6th month of the second pregnancy, a first panic attack occurred, and physicians suspected it was a pulmonary embolism. They ordered computed tomography angiography (CTA) in the 7th month. Following this intervention, she looked up information on Google and discovered a recognizable relationship between the procedure and a hazardous pregnancy situation. The individual did not have any medical issues and passed through natural growth. Her mother took on most of their tasks because her father's work required him to travel to numerous places and overseas. According to the patient's disclosure, her mother was an anxious person; for instance, she locked all the doors whenever she intended to leave the house. According to this patient, she was worried and obsessed at school and felt like a failure if she received low exam scores. In addition, the patient was reliant on her family and nevertheless preferred to leave the university due to its distance from them. She had a negative family history, and there was not enough evidence to prove a positive familial history related to Parkinsonism. However, 2 years before this presentation, she experienced more anxiety than usual. She was more dependent on her mother because she felt sorrow about the demise of her father. An appealing emotional relationship did not define her married life.

## 3 Methods (Differential Diagnosis, Investigations, and Treatment)

Following her physical examination, the patient appeared apprehensive, with slight shaking in her extremities and a type of hand tremor increased by attention seeking and lessened by distractibility. There was also no discernible pattern to the tremor. There were no rigidities or irregularities in this person's gait. Bradykinesia was absent, and the glabellar sign was negative. According to her history and physical examination, it seemed she had a faking bad and a pessimistic ideation. Although there was a slight postural tremor in her hands, there were no actual indications of Parkinson's disease found during the physical examination, while at rest tremor has a potential connection to Parkinson's disease. The results of the physical examination are written in (Table 1). After examining the patient, a neurologist and a neuropsychiatrist specialist concluded that the patient had no movement abnormalities. The individual received supportive treatment and an explanation of the etiologies of the condition. She was given medications to reduce her stress and anxiety. Amantadine and Levodopa administration were prevented. She was recommended to avoid excessive laboratory testing, unnecessary paraclinical evaluations, and frequent visits to various doctors. Organizing follow‐up visits at intervals of 1–2 months was advised. The patient was also reassured that there is no evidence of a severe, life‐threatening, or challenging‐to‐treat condition. It is advantageous when physicians take time to explain this disorder in detail, especially the condition that the patient fears developing. Furthermore, giving reassurance that these symptoms and physical examinations do not correlate to Parkinson's disease should be a priority. Moreover, we should advise the patient to prevent repeat appointments with physicians and unnecessary tests or scans. Besides, we should try to relieve the patient from stress and her mind conflicts; additionally, SSRIs and TCAs are the best choices, avoiding anti‐Parkinsonism consumption, and supportive treatment such as cognitive behavioral therapy (CBT) would come with advantages. One‐month follow‐up durations are considered to be useful.

**TABLE 1:** Neurological examination findings.

Parkinsonism‐related examination	Clinical findings
Myerson's sign (glabellar tap test)	Negative
Bradykinesia	Negative
Gait	Normal
Rigidity	Negative
Muscle tone	Normal
Finger tapping test	Normal
Tremor at rest	Negative
Postural tremor	Positive
Anosmia or smell abnormalities	Negative
Digit Span	Normal
Curved posture	Negative
Masked Face	Negative
Cerebellar's signs	Negative
Hypophonia	Negative

Because of rest tremor and other Parkinson's signs being absent, on the other hand, postural tremor was dominant, and she was so anxious and suggestible with cluster C personality. Primary Parkinsonism or Parkinson‐plus syndrome was ruled out, which is why illness anxiety disorder is the first diagnosis. The second diagnosis is major depressive disorder (MDD); besides IAD, the third differential diagnosis is Functional neurological disorder (FND). Functional Parkinsonism might be found if unexpected shaking and other symptoms occur at the onset [14]. In FND, the patient may experience Parkinsonism symptoms while not being concerned about developing Parkinson's disease; the scenario is different in our case because the main problem that occupies the major portion of this patient's mind is the fear of Parkinsonism. The fourth distinction is the use of herbal or industrial drugs such as Theophylline or Salbutamol. The fifth one is anticipated if the patient's primary gain was to obtain a disability certificate or aim to attract attention as a secondary gain related to malingering or factitious disorder. We did not find any evidence supporting primary or secondary gain in this case, which is why factitious disorder and malingering were ruled out.

## 4 Conclusion and Results (Outcome and Follow‐Up)

A patient with illness anxiety disorder may present to a physician with Parkinson's‐like symptoms, and there is limited data and reports on this matter. The psychiatrist, while needing to examine the patient thoroughly, must also provide the necessary information about the disorder to the patient and avoid requesting unnecessary interventions and paraclinical approaches.

## 5 Discussion

In illness anxiety disorder (IAD), patients insist they have a specific disease or claim they will soon develop a serious illness [15]. Individuals with IAD, compared to the healthy population, demonstrated fewer symptoms, but a major portion of their attributions they generated were somatic illness, with more severe symptoms in their intensity [16]. The patient insisted she had Parkinson's disease. After a neurological examination, we found no sign or symptom related to Parkinson's disease. No symptoms, including bradykinesia, slowness, rigidity, curved posture, masked face, or dysarthric speech, were observed. The only type of tremor noted was mild and physiological in the upper limbs, resulting from stress. The patient's age and the progression of the disease did not match with Parkinson's disease. The mentioned findings ruled out a diagnosis of Parkinson's disease.

According to the psychiatric evaluation, she reported family problems and marital discords, anxiety, fear of illness, and significant preoccupation with illness, as well as mild depression. She did not have primary gain. Additionally, the lack of willingness to be hospitalized also indicates a rejection of secondary gain and factitious disorder, so the diagnosis of malingering was excluded. Unlike Parkinson's patients who do not seek frequent medical visits for confirmation of their illness, the patient is seeking validation of their suspicions about their condition and frequently visits different physicians. Based on the available evidence, the disease diagnosis could be illness anxiety disorder (IAD). There are similar cases regarding the presence of this disorder alongside other neurodegenerative diseases such as multiple sclerosis, but this is the first instance of this disease associated with concern about Parkinson's disease.

Factors such as growing up in a strict family regarding health matters, experiencing similar and severe illnesses in childhood, and observing similar conditions in close relatives can contribute to the development of this disorder [6]. After a distant relative experienced tremor and subsequently passed away, the patient had come to believe that Parkinson's disease was the cause of their death and feared that she too would soon develop this illness, ultimately leading to disability and death. The anxiety stemming from this had manifested as symptoms that the patient interprets as Parkinson's disease. Typically, these patients present with concerns about diseases such as multiple sclerosis, human immunodeficiency virus (HIV), hepatitis, and cancer; in addition, rare reports of this disease have also been observed in the context of pulmonary thromboembolism and rabies [10, 11]. However, this patient had specifically referred to Parkinson's disease.

Treatment of the disorder is usually carried out by supporting and assisting patients in controlling anxiety and stress and coping with existing problems and conflicts [3].

The patient was informed about the nature of Parkinson's disease and how the symptoms appeared and intensified. Antidepressant medications were prescribed, but initially, the patient refused to take the medicines due to the emphasis on having Parkinson's disease. Eventually, the patient agreed to take the medications, and after receiving them in follow‐up appointments 3 weeks later, the patient's symptoms improved and the disease subsided.

## Author Contributions

**Ronak Fatahi:** writing – original draft, writing – review and editing. **Mohammad Sadegh Khooshab:** writing – original draft, writing – review and editing. **Seyede Fatemeh Sadatakhavi:** writing – original draft. **Amin Ziyaei:** writing – original draft, writing – review and editing. **Reza Bidaki:** conceptualization, data curation, supervision.

## Ethics Statement

The human studies were approved by the Ethics Committee of Shahid Sadoughi University of Medical Sciences, Yazd, Iran (Approval code: IR.SSU.MEDICINE.REC.1403.338).

## Consent

The patient gave her full consent to write this paper and made the necessary contributions; as she said, “I give my full consent for writing a scientific paper about my disease”. Written informed consent was obtained from the patient to publish this report in accordance with the journal's patient consent policy.

## Conflicts of Interest

The authors declare no conflicts of interest.

## Data Availability

The details of contributions are listed in the article; other information can be directed to the corresponding author.
